# Transthyretin amyloidosis in patients with spinal stenosis who underwent spinal surgery: a systematic review and meta-analysis

**DOI:** 10.3389/fneur.2024.1425862

**Published:** 2024-10-09

**Authors:** Domantas Tamasauskas, Laura Tamasauskiene

**Affiliations:** ^1^Department of Neurosurgery, Lithuanian University of Health Sciences, Kaunas, Lithuania; ^2^Department of Immunology and Allergology, Lithuanian University of Health Sciences, Kaunas, Lithuania; ^3^Laboratory of Immunology, Department of Immunology and Allergology, Lithuanian University of Health Sciences, Kaunas, Lithuania

**Keywords:** ATTR, amyloidosis, spinal stenosis, lumbar stenosis, ligamentum flavum

## Abstract

**Background:**

Accumulation of transthyretin amyloids (ATTR) is detected in ligamentum flavum in about 1/3 of patients underwent surgery for spinal stenosis. However, the significance of this finding is not known. The aim of this systematic review and meta-analysis is to analyze the incidence and importance of ATTR in patients with spinal stenosis who underwent spinal surgery.

**Methods:**

The primary outcome measure was incidence of ATTR in patients with spinal stenosis. English language observational studies published within 10 years period were searched in Pubmed and Taylor and Francis databases.

**Results:**

Nine articles were included in the systematic review. The incidence of positive ATTR among patients who underwent lumbar spinal surgery was 48% (95%CI 38–58%). ATTR deposits were found in the lumbar region the most frequently. Seven studies showed that patients with positive ATTR were older than those with negative. Five studies investigated and found a significant relationship between the ligamentum flavum thickness and positive ATTR. Five studies investigated cardiac involvement among patients with positive ATTR.

**Conclusion:**

ATTR deposits are frequently found in older patients with spinal stenosis, especially in the lumbar region. The presence of ATTR deposits is related to ligamentum flavum thickness.

## Introduction

Amyloidosis is a disorder of protein accumulation, during which the normal architecture and function of the tissue in which the protein accumulates is disturbed (1). More than 36 amyloid precursor proteins are known. Depending on the amount and localization of amyloid deposits, the spectrum and severity of diseases varies.

Transthyretin (TTR), a serum protein synthesized mainly in the liver, causes two types of systemic amyloidosis. Wild-type transthyretin amyloidosis (ATTR) (or senile systemic amyloidosis) is a non-hereditary form of amyloidosis caused by dissociation and misfolding of serum protein transthyretin (1). Wild-type ATTR is a normal genotype of TTR. Another type is a hereditary systemic amyloidosis, which is associated with abnormal genetic sequence (variant TTR). Wild-type ATTR primarily manifests as cardiomyopathy while ATTR due to a genetic variant manifests as cardiomyopathy and/or polyneuropathy (1). Moreover, they are also found in tendons, ligaments, and joints, particularly in older individuals (1, 2). For example, accumulations of wild-type ATTR are detected in ligamentum flavum in 33–45% of patients underwent surgery for spinal stenosis (2).

Spinal stenosis is defined as the narrowing of the spinal canal causing clinical symptoms such as numbness, fatigue and/or pain in the neck, buttocks and/or legs that increase with activities such as walking and standing (3–5). Spinal stenosis can involve the cervical, thoracic, or lumbar spine, being either monosegmental or multisegmental, and unilateral or bilateral (4). Lumbar spinal stenosis is the most frequent form and according to the systematic review and meta-analysis its incidence varies from 11 to 39% (3). Symptomatic lumbar spinal stenosis is characterized by low back and leg pain in the setting of compression of the central canal and/or exiting nerve roots by disk, osteophyte, ligamentum flavum, or other structures (5). Spinal stenosis usually develops due to many reasons such as degenerative diseases, iatrogenic factors, trauma, metabolic, infectious or rheumatological diseases (4). Treatment of spinal stenosis can be conservative (nonsteroidal anti-inflammatory medications, acetaminophen, and other medications, physical therapy, epidural steroid and local anesthetics injections) and surgical (spinal decompression surgery) (5). Direct surgical decompression, in which bone and/or disk are moved away from the affected nerve root(s), can be performed through an open or minimally invasive approach for lumbar spinal stenosis. In patients with concomitant degenerative spondylolisthesis and/or scoliosis, decompression for lumbar spinal stenosis is often performed in combination with lumbar arthrodesis (fusion), in which adjacent vertebrae are fused to prevent motion (5).

The clinical relevance of ATTR in ligamentum flavum is not fully understood. Scientists investigate whether ATTR depositions in spinal cord could be related to outcomes of spinal stenosis and prevalence of other disorders such as heart damage or systemic amyloidosis (6, 7).

The aim of this systemic review is to analyze the incidence and importance of ATTR in patients with spinal stenosis who underwent spinal surgery.

## Methods and materials

### Measured outcomes

Incidence of amyloidosis in patients with spinal stenosis was the exposure of interest. The primary outcome measure was incidence of ATTR in patients with spinal stenosis. Secondary outcomes of interest included: localization of amyloid deposits, relation between ligamentum flavum thickness and amyloid deposits, relation between amyloid deposits and systemic amyloidosis and/or another local amyloidosis (such as carpal syndrome or cardiac amyloidosis). Data about the number of participants and their age was also collected.

### Eligibility criteria

English language observational studies (cross-sectional) were included if they reported incidence of ATTR in patients with spinal stenosis published within the period of 2012–2022 years.

### Search strategy and statistical analysis

Studies were searched in Pubmed and Taylor and Francis databases. The Medical Subject Heading (MeSH) terms of “spinal stenosis” or “ligamentum flavum” and “amyloid” or “amyloidosis” were used. In addition, combined text words of (spinal stenosis AND amyloid) OR (spinal stenosis AND amyloidosis) OR (ligamentum flavum AND amyloid) OR (ligamentum flavum AND amyloidosis) were used to find relevant studies. Systematic review was performed according to the PRISMA guidelines (8). Statistical heterogeneity was assessed using the *I*^2^ statistic. We conducted a random-effects meta-analysis by the DerSimonian and Laird method. Before pooling, the Freeman-Tukey double arcsine transformed proportion was used to ensure admissible CIs given its improved estimation of CIs in the presence of 0 events and its more stable estimation of variances. The exact method was used for CI computation for pooled estimation of prevalence and incidence.

## Results and discussion

### Study characteristics

**Figure 1:**
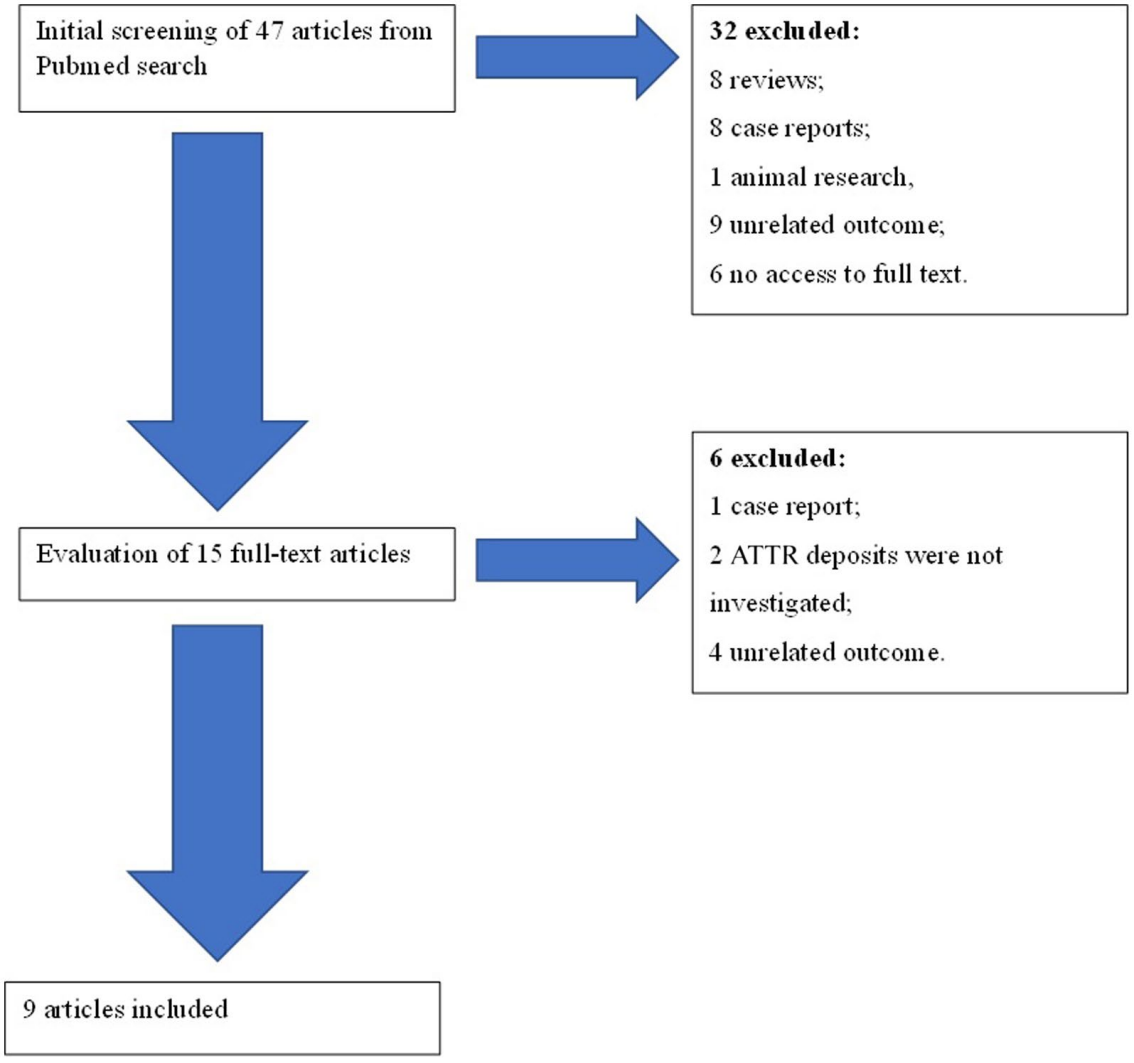
Chart of studies included in the systematic review.

A total of 47 articles were found. Of these articles, 32 were eliminated after an initial screening of their titles and abstracts (Figure 1). The full texts of the 15 remaining articles were assessed in detail. Six articles were rejected. Finally, nine articles were included in the systematic review. Articles were included after evaluation of their quality according to Quality Assessment Tool for Observational Cohort and Cross-Sectional Studies of National Heart, Lung and Blood Institute (Table 1) (9).

**Table 1:** Quality Assessment Tool for Observational Cohort and Cross-Sectional Studies of National Heart, Lung and Blood Institute.

Study	Quality
Westermark P., et al., Upsala Journal of Medical Sciences, 2014	Fair
Yanagisawa A., et al., Modern Pathology, 2015	Good
George KM., et al., World Neurosurgery, 2020	Good
George KM., et al., Clinical Neurology and Neurosurgery, 2021	Good
George KM., et al., Journal of Clinical Neuroscience, 2021	Good
Eldhagen P. et al., Journal of Internal Medicine, 2021	Good
Wang AY., et al., World Neurosurgery, 2022	Good
Maurer MS., et al., Journal of the American Geriatrics Society, 2022	Fair
Yaseen MA., et al., Cureus, 2022	Fair

### Measurement of ATTR and ligamentum flavum thickness

Amyloidosis in pathologic specimens was detected with Congo red staining and demonstration of apple-green birefringence under polarized light and in all studies. Wild type ATTR was confirmed by the presence of wild type ATTR by typing and the absence of mutations in the ATTR gene sequence from genomic DNA in three studies (10–12). Wild type ATTR was detected using monoclonal antibodies to ATTR in four studies (13–16). Tandem mass spectrometry-based analysis was used in two studies (17, 18).

Ligamentum flavum thickness was measured using an axial T2-weighted magnetic resonance images (MRI) slice in three studies (11, 12, 18) and using an axial T1- weighted MRI in two studies (13, 15). Three studies indicated that bilateral measurements were made (11, 12, 18). The average of the three measurements of MRI images was taken as a final value in two studies (12, 15) and the arithmetic mean of the two values was recorded in one study (11). Ligamentum flavum thickness was measured in affected lumbar levels, but lumbar Ligamentum flavum burden was calculated as a mean Ligamentum flavum thickness from each lumbar level in two studies (12, 18).

### Measurement of cardiac involvement

Five studies evaluated cardiac involvement. Three studies evaluated only clinical symptoms of cardiac failure, hypertension, arrhythmia and myocardial infarction usually based on history (10, 11, 15). Eldhagen et al. (14) performed cardiovascular investigation with electrocardiography, medical history, physical examination, N-terminal pro–B-type natriuretic peptide (NT-proBNP), echocardiography with strain analysis and cardiovascular MRI (CMRI; Siemens 1 5 T) with gadolinium contrast for patients with positive ATTR deposits. Yaseen et al. (13) also performed cardiac investigation which involved medical history, physical examination, NT-proBNP, electrocardiography, echocardiography and Tc-99 m pyrophosphate planar imaging with concomitant single photon emission computed tomography (SPECT).

### Measurement of carpal tunnel syndrome

Four studies evaluated carpal tunnel syndrome. Information about carpal tunnel symptoms, surgery or muscle tears was obtained from chart review or history in all these studies (10–12, 14).

### Studies populations

Patients who underwent lumbar spinal surgery were involved in all studies. Spinal stenosis as indication for surgical treatment was in all studies. Four studies additionally involved patients with disk herniation (10–12, 15) and one study—with disk degeneration (13). Overall, 1,339 patients underwent investigation for ATTR deposits in ligamentum flavum. The average patients’ age was about 70 years.

### Incidence of ATTR in patients with spinal stenosis

**Figure 2:**
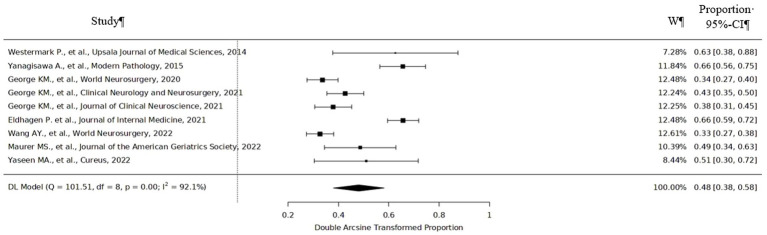
Forest plot of studies on incidence of ATTR in patients with spinal stenosis.

According to meta-analysis, the incidence of positive ATTR among patients who underwent lumbar spinal surgery was 48% (95%CI 38–58%) varying from 33% (95%CI 27–38%) to 66% (95%CI 56–75%) (Figure 2). ATTR deposits were found in the lumbar region the most frequently. 70% specimens that were ATTR positive were found to occur in the L3-L4 and L4-L5 levels (8). One study showed that 24 (89%) patients had ATTR deposits in the lumbar region, and 3 (11%) patients had ATTR deposits outside of the lumbar spine (2 had ATTR in the cervical level, while 1 had deposition in the thoracic level) (10). Only three positive ATTR cases were found among patients with disk herniation and no ATTR deposits among patients with lumbar disk degeneration.

There was no significant difference between females and males in all studies. Seven studies showed that patients with positive ATTR were older than those with negative. Westermark et al. (16) noticed a tendency that patients with ATTR positive amyloid deposits were older than patients with ATTR negative findings (79.0 ± 5.6 vs. 58.1 ± 9.3 years). Yanagisawa et al., George et al., George et al., and Maurer et al. provided statistically significant results that patients’ with positive ATTR average age is higher compared with those without ATTR deposits (11, 12, 15, 17). Eldhagen et al. (14) found that ATTR was significantly more prevalent in the 70- to 79-year age group than in the younger age groups. Yaseen et al. (13) showed that the results were positive for ATTR in the 51–60 and 61–70 age groups only, and negative in all other younger age groups. One study revealed that patients older than 70 were 4.8 times more likely to have amyloid in the ligamentum flavum (11).

### Relation between ligamentum flavum thickness, systemic amyloidosis and/or other local amyloidosis and amyloid deposits in spinal cord

Five studies investigated and found significant relationship between the ligamentum flavum thickness and positive ATTR (11–13, 15, 18). Only one study investigated correlation between symptoms and quality of life and ATTR deposits but found no significant results (17). There was no statistically significant difference in the number of spinal levels that required operation between the patients with positive ATTR and patients with negative ATTR (11).

Five studies investigated cardiac involvement among patients with positive ATTR, three of them found cardiological abnormalities, but signs of cardiac amyloidosis were not found (10, 11, 14, 15, 17). Three studies noticed carpal tunnel syndrome among patients with positive ATTR (10–12, 14). One study showed that carpal tunnel syndrome was more frequent in patients with positive ATTR compared to patients with negative ATTR (12).

Summary of the results of reviewed articles are presented in Table 2.

**Table 2:** Summary of the main results of reviewed studies.

Study	Population/samples	Prevalence of positive ATTR amyloidosis deposits	Difference of positive ATTR deposits between age groups	Relation between ATTR deposits and ligamentum flavum thickness	Abnormalities in cardiovascular system in patients with positive ATTR	Presence of carpal tunnel syndrome in patients with positive ATTR
Westermark P., et al., Upsala Journal of Medical Sciences, 2014	15 patients with lumbar spinal stenosis	5 (33.3%; 2 women, 3 men), age 79.0 ± 5.6 years	Patients with TTR positive amyloid deposits tended to be older than patients with TTR negative findings (79.0 ± 5.6 vs. 58.1 ± 9.3 years)	No data.	No data.	No data.
Yanagisawa A., et al., Modern Pathology, 2015	95 specimens from 56 patients with lumbar spinal canal stenosis 21 specimens from 19 patients with lumbar disk herniation.	Spinal canal stenosis: 43 (45.3%; 17 women, 26 men), age 74 ± 7.6 years. Lumbar disk herniation patients 0 (0%)	Patients with ATTR positive amyloid deposits were older than patients with ATTR negative findings (74 ± 7.6 vs. 68 ± 6.4 years, *p* < 0.0001)	Higher thickness of ligamentum flavum was observed in patients with ATTR positive amyloid deposits than patients with ATTR negative findings (4.36 ± 0.79 vs. 3.39 ± 0.75 mm, *p* < 0.0001)	No cardiac involvement.	No data.
George KM., et al., World Neurosurgery, 2020	251 patients with spinal stenosis and disk herniation	27 (10.8%; 11 women, 16 men), age 71 (interquartile range 9) years. With spinal stenosis 26 (96.3%) With disk herniation 1 (3.7%)	No data	No data	Hypertension was present in 23 (85%) patients. Eight (30%) patients had a history of cardiac symptoms: Heart failure in 2, Arrhythmia in 4, and Myocardial infarction in 2.	Carpal tunnel release was performed for 5 patients.
George KM., et al., Clinical Neurology and Neurosurgery, 2021	177 patients (69 women, 108 men), age 64.8 ± 12.0: Lumbar spinal stenosis in 163 Lumbar disc herniation in 14	30 (16.9%; 10 women, 20 men), age 72.6 ± 7.6 years: With spinal stenosis 29 (96.7%) With disc herniation 1 (3.3%)	Patients with ATTR were older than patients without ATTR (72.6 ± 7.6 vs. 63.2 ± 12.1, *p* < 0.001).	Ligamentum flavum was thicker in patients with ATTR deposits than in patients without ATTR (4.64 vs. 3.99 mm, *p* < 0.001). Presence of ATTR increased lumbar ligamentum flavum burden by 2.63 mm (95% confidence interval (CI): 0.44–4.82 mm, *p* = 0.02) when adjusted for age.	No data.	In patients with ATTR amyloid carpal tunnel syndrome was more frequent than in patients without ATTR (20% vs. 6.8%, *p* = 0.022)
George KM., et al., Journal of Clinical Neuroscience, 2021	178 (women 69, men 109), age 64.0 ± 11.6 Lumbar spinal stenosis 161 Lumbar disc herniation 17	24 (13.5%; 9 women, 15 men), age 64.0 ± 11.6 With spinal stenosis 23 (95.8%) With disc herniation 1 (4.2%)	The average age at surgery was higher in patients with ATTR than in patients without ATTR (71.3 ± 6.8 vs. 62.9 ± 12.1 years, *p* = 0.001).	The mean thickness of ligamentum flavum was higher in patients with ATTR than in patients without ATTR (4.92 ± 1.27 vs. 4.00 ± 1.21 mm, *p* < 0.001).	Hypertension in the ATTR positive group was more frequent than in patients without ATTR (87.5% vs. 60.4%, *p* = 0.010)	No difference of carpal tunnel release between groups.
Eldhagen P. et al., Journal of Internal Medicine, 2021	250 patients with lumbar spinal stenosis (142 women, 108 men), age 67.8 ± 8.0 years	93 (37.2%)	ATTR amyloidosis was more prevalent in the 70- to 79-year age group than in the younger age groups in both women (*p* < 0.0001) and men (*p* < 0.002).	No data	Atrial fibrillation in 1 patient. Ischaemic heart disease in 1 patient. Aortic valve disease in one patient. No signs of cardiac amyloidosis	Five men had a history of carpal tunnel syndrome.
Wang AY., et al., World Neurosurgery, 2022	324 patients with lumbar spinal stenosis	33 (10.19%; 13 women, 20 men) age 72.7 ± 7.2 years	No data	Amyloid load was shown to be positively correlated with ligamentum flavum thickness (*R* = 0.38, *p* = 0.02).	No data.	No data.
Maurer MS., et al., Journal of the American Geriatrics Society, 2022	47 patients with lumbar spinal stenosis (22 women, 25 men), age 68 ± 5 years.	10 (21,28 proc., 2 women, 8 men), age 72.4 ± 8 years	Patients with ATTR tended to be older than patients without ATTR (72.4 ± 8 vs. 67.8 ± 6.1 years).	No data	One patient had cardiac involvement.	No data
Yaseen MA., et al., Cureus, 2022	22 (10 women, 12 men), age: 46 (range 25–70) years: 10 patients with lumbar spinal stenosis 11 patients with lumbar disc degeneration	With spinal stenosis 5 (22%) With lumbar disk degeneration 0 (0.0%)	The results were positive for ATTR in the 51–60 and 61–70 age groups only, while negative in all other younger age groups	Mean LF thickness was higher in ATTR positive group compared to ATTR negative group (4.64 ± 0.16 vs. 3.21 ± 0.81 mm, *p* = 0.001)	No data	No data

## Conclusion

ATTR deposits, especially wild type ATTR, are frequently found in older patients with spinal stenosis, especially in the lumbar region, as is the case with wild type ATTR anywhere. The presence of ATTR deposits is related to ligamentum flavum thickness. Moreover, patients with positive ATTR tend to have cardiac abnormalities and tunnel carpal syndrome, but more evidence is needed. However, there is lack of evidence about the impact of ATTR in ligamentum flavum on the severity of symptoms of spinal stenosis, quality of life, number of spinal levels that require surgery, motor function, gait stability, fall frequency and independent ambulation in an elderly patient population post operatively.

## Data Availability

The original contributions presented in the study are included in the article/supplementary material, further inquiries can be directed to the corresponding author.
